# Reduced number of endothelial progenitor cells in adult patients with beta thalassemia major

**DOI:** 10.1007/s00277-026-06979-1

**Published:** 2026-04-14

**Authors:** Idan Goldberg, Idit Pazgal, Pinhas Stark, Arza Steimatzky, Dorit Leshem Lev, Ori Tishler, Tzippy Shochat, Pia Raanani, Ran Kornowski, Galia Spectre

**Affiliations:** 1 Institute of Hematology, Beilinson Hospital, Rabin Medical Center, Petah Tikva, 49100 Israel; 2 Gray Faculty of Medical and Health Sciences, Tel Aviv University, Tel Aviv, Israel; 3 Comprehensive Center of Thalassemia, Hemoglobinopathies & Rare Anemias, Institute of Hematology, Beilinson Hospital, Rabin Medical Center, Petah Tikva, Israel; 4 Felsenstein Research Center, Rabin Medical Center, Petah Tikva, Israel; 5 Department of Internal Medicine F - Recanati, Beilinson Hospital, Rabin Medical Center, Petah Tikva, Israel; 6 Statistical Consulting Unit, Beilinson Hospital, Rabin Medical Center, Petah Tikva, Israel; 7 Department of Cardiology, Rabin Medical Center, Petah Tikva, Israel

**Keywords:** Endothelial progenitor cells, Beta thalassemia major, Hypercoagulable state, Colony-forming units, Endothelial dysfunction

## Abstract

Beta-thalassemia major (TM) is associated with a high incidence of thromboembolic events and prothrombotic changes, suggesting a chronic hypercoagulable state. Hematopoietic derived endothelial progenitor cells (hEPCs), derived from the bone marrow, contribute to vascular repair, and their quantity and function inversely correlate with thrombotic risk. In this study, we aimed to evaluate the number and colony-forming capacity of hEPCs in adult patients with TM. hEPCs were isolated from peripheral blood mononuclear cells of TM patients and healthy controls. Flow cytometry was used to measure the proportion of mononuclear cells co-expressing VEGFR-2 and CD34 or CD133. hEPCs were cultured for 7 days, after which colony-forming capacity and viability were assessed via microscopy and MTT assay. TM patients (*n* = 25) had a significantly lower proportion of VEGFR-2^+^CD34^+^ cells compared to controls (*n* = 11); 0.95% (0.36–1.65%) vs. 1.78% (1.1–4.2%), *p* = 0.02. hEPC colony counts and viability were also reduced: 0 (0–0.5) vs. 3 (2–4), and 0.02AU (0.01–0.05) vs. 0.2AU (0.18–0.23), respectively (both *p* < 0.0001). Subgroup analyses suggested lower VEGFR-2^+^CD34^+^ proportions in patients with prior thrombosis (*n* = 6) and in splenectomized patients (*n* = 21). Patients with TM exhibit reduced hEPC numbers and impaired colony-forming capacity, which may be associated with the hypercoagulable state observed in this population.

## Introduction

Beta Thalassemia is an inherited blood disorder characterized by impaired beta globin production, ineffective erythropoiesis, reduced red blood cell survival, and subsequent hemolytic anemia [1–3]. Individuals diagnosed with beta thalassemia major (TM) require lifelong blood transfusions beginning in early childhood and are burdened by significant health complications and reduced life expectancy [1, 4–7]. In recent decades, advances in treatment have led to a marked improvement in survival among patients with TM; as a result, this increased longevity has been accompanied by growing evidence of long-term complications, including both venous and arterial thromboembolic events [4, 6, 8–13].

Both patients with thalassemia major and those with thalassemia intermedia are known to exhibit a hypercoagulable state, manifested by an increased risk of both venous and arterial thromboembolic events [8, 12–15], including a high incidence of ischemic lesions among these patients [10]. According to two recent studies, including a work from our group, 40–60% of TM patients had silent ischemic brain lesions on MRI [11, 16]. Several prothrombotic factors were previously identified among TM patients: increased platelet activation and aggregation [17–20], deficiency of natural anticoagulants [17, 21–23], inhibition of fibrinolysis [24], and pathological red blood cells [18, 25].

Endothelial progenitor cells (EPCs) comprise a heterogeneous group with the capacity to differentiate into endothelial cells, contributing to neovascularization as well as vascular repair. Emergent evidence indicates that EPCs may also be involved in modulating inflammatory response. Circulating EPCs include two main subpopulations: (a) hematopoietic-derived EPCs (hEPCs), which originate in bone marrow and exert their primary effects through paracrine signaling and (b) non-hematopoietic EPCs, which exhibit a greater capacity for direct endothelial integration and vessel formation [26]. hEPCs, which are mobilized by a range of cytokines following vascular injury, participate in the restoration and regeneration of damaged endothelium [27, 28]. Importantly, both reduced numbers and impaired function of circulating hEPCs have been linked to an elevated risk of thrombosis [27, 29–33].

hEPCs were shown to be suppressed in anemic patients with acute coronary syndrome [34]. However, to the best of our knowledge, the number and colony-forming capacity of hEPCs in TM patients have only been evaluated in one previous study in adults [35].

We hypothesized that patients with TM have reduced numbers of hEPCs, which may contribute to their thrombotic tendency. Accordingly, we assessed the quantity and colony-forming capacity of hEPCs in TM patients.

## Methods

### Study population

The study population included TM patients, > 18 years of age, followed and treated in our thalassemia clinic, regularly transfused every 2–3 weeks since early childhood. These patients were included in our previous reported study that assessed the incidence of silent cerebral infarcts (SCI) by T2-brain magnetic resonance imaging (MRI) [11]. Exclusion criteria were estimated creatinine clearance below 30 ml/min or participation in another clinical trial. The study population also included healthy volunteers, age-matched, that had no chronic illnesses and no regular medications, as normal controls.

Approval for the study was granted by the Institutional Review Board of Rabin Medical Center. It was carried out in accordance with the ethical guidelines for human research established by the responsible committee, and adhered to the principles of the Declaration of Helsinki (1975, revised 2008). Informed consent, in written form, was secured from all patients and controls prior to participation.

The patients’ medical charts were reviewed to obtain clinical and laboratory data. Clinical data included age, gender, history of splenectomy, history of cardiovascular risk factors, and current iron chelation therapy. Laboratory data included hematologic data, blood chemistry data and serum ferritin levels. Imaging data included MRI T2* studies of the brain retrieved from our previous study [11].

### Laboratory methods

All endothelial cell measurements were performed by a single experienced laboratory investigator using standardized and routinely applied laboratory methods, ensuring procedural consistency. The investigator has extensive experience with this technique and has conducted multiple prior studies employing the same methodology. Importantly, the investigator was blinded to the clinical origin of the samples, thereby minimizing potential measurement bias.

#### hEPC isolation and quantification

Peripheral blood was drawn before routine transfusion, and peripheral blood mononuclear cells (PBMCs) were isolated via Ficoll gradient. After lysing red blood cells, hEPC levels were measured as previously described [31, 36, 37]. Briefly: isolated cells were stained with FITC-labeled VEGFR-2 (vascular endothelial growth factor receptor-2), a key endothelial lineage marker (R&D, Minneapolis, USA), CY5.5 labeled CD45 (Dako, Denmark), and either PE labeled CD133 or CD34 (Miltenyi Biotech, Auburn, CA, USA). Isotype-identical antibodies were used as controls. Samples were analyzed by flow cytometry (FACSCalibur, Becton Dickinson), acquiring 100,000 events gated on CD45⁺ cells (excluding debris). A broad mononuclear gate was applied based on forward and side scatter characteristics after exclusion of debris and cell fragments; analyses were then performed within CD45⁺ PBMCs. Within this population, CD34⁺ or CD133⁺ cells were assessed for VEGFR-2 co-expression, ensuring these progenitor cells are of endothelial origin. Analyses were performed in duplicate, and results reported as the percentage of CD45⁺ PBMCs co-expressing VEGFR-2 with CD34 or CD133.

#### Colony forming unit (CFU) quantification

Isolated PBMCs were resuspended in Medium 199 (Invitrogen, Carlsbad, CA, USA) supplemented with 20% fetal calf serum (Gibco BRL Life Tech, Gaithersburg, MD, USA) and plated (5 × 10⁶ cells/well) on fibronectin-coated 6-well plates. These culture conditions are designed to selectively support the growth of endothelial progenitor cells, thereby enabling their isolation from the total PBMC population. After 7 days, hEPC colonies (defined as ≥ 100 flat cells surrounding a cluster of rounded cells) were counted using inverted microscopy [31, 36, 37]. Central clusters without surrounding cells were excluded. Endothelial identity of colonies was confirmed in our previous studies using immunostaining of randomly selected colonies with antibodies directed against VEGFR-2, CD31 (Becton Dickinson, NJ, USA), and Tie-2 (Santa Cruz, California, USA) [36]. In the present study, colony counts are reported as the mean CFU) number per field.

#### Viability of hEPC colonies

Prior to plating, cell viability was confirmed by Trypan Blue exclusion to ensure that only viable cells were seeded. Cell viability was further assessed using the MTT assay kit (Sigma, St. Louis, USA). Briefly, after 7 days of culture, hEPCs were incubated with MTT (1 mg/mL) for 3–4 h. Viable cells reduced MTT to insoluble formazan crystals, which were solubilized in isopropanol and quantified by absorbance at 570 nm (reference 690 nm). Optical density was directly proportional to the number of viable cells.

#### Mixing study

In order to detect the effect of patients’ plasma on CFU growth, plasma obtained from TM patients (40%) was added to PBMCs of healthy volunteers’ culture media (*n* = 3) in the presence of medium 199 (40%) and fetal calf serum (20%). The ability to perform CFU in this mixing study was compared to PBMCs from the same controls that were suspended with medium 199 (80%) and fetal calf serum (20%). CFU quantification and viability of hEPC colonies were measured as described above.

#### Endothelial related signaling molecules

The levels of different endothelial related pro-angiogenic and inflammatory mediators were measured by ELISA assays according to the manufacturer’s recommendations, including vascular endothelial growth factor (VEGF), stromal cell-derived factor 1 (SDF-1) and p-selectin (Bio-Techne, Minneapolis, Minnesota).

### Statistical methods

Statistical analyses were performed using SAS software, version 9.4. Continuous variables are summarized as medians with interquartile ranges (IQR), while categorical variables are reported as counts and percentages (n, %). The Kolmogorov–Smirnov test was applied to evaluate the distribution of continuous variables. Group comparisons were conducted using the independent samples t-test for normally distributed continuous variables, the Wilcoxon rank-sum test for non-normally distributed variables, and Fisher’s exact test for categorical variables. A two-sided p-value < 0.05 was considered indicative of statistical significance.

## Results

### Patients’ characteristics

Twenty-five patients were included in the study. Their median age was 35 (31–40) years and 13 (52%) were females. Most of the patients (84%) were splenectomized (SPX). All TM patients were treated with standard iron chelation therapy (ICT); 4 patients with deferoxamine (DFO), 4 with deferiprone (DFP), 8 with deferasirox (DFX), and 9 with a combination of deferoxamine and deferiprone (DFO + DFP). Fifteen healthy volunteers, matched for age and gender, were included as normal controls. Due to sample availability, flow cytometry and CFU/MTT analyses included 11 controls (as indicated in figure legends).

An overview of the clinical and laboratory findings is provided in Table 1.

**Table 1:** Clinical and laboratory characteristics of the study group

Parameter	TM patients *n* = 25	Controls^*^ n = 15
Age (years)	35 (31–40)	35 (34-36.75)
Female (n, (%))	13 (52%)	7 (46%)
Hemoglobin (g/dL)	9.7 (9.1–10.1)	14.4 (12.85–15.42)
WBC (x10^9^/L(	14.6 (10.9–18.9)	6.8 (5.6–7.6)
Platelet count (x10^9^/L(	522 (391–619)	246 (224–274)
Ferritin (ng/ml)	1111 (286–2265)	NA
Number of focal bright foci on brain MRI^†^	3 (0–7)	NA
History of splenectomy	21 (84%)	0
History of VTE	5 (20%)	0
History of ATE	1 (4%)	0
Aspirin treatment	7 (28%)	0
Anticoagulation treatment	2 (8%)	0
Cigarette smokers	7 (28%)	NA
Diabetes mellitus	4 (16%)	0
Type of chelation therapy		
Deferoxamine	4 (16%)	
Deferasirox	8 (32%)	
Deferiprone	4 (16%)	
Combination treatment of Deferoxamine and Deferiprone	9 (36%)	

Values are in median (IQR)

* Controls: *n* = 15 for baseline characteristics; *n* = 11 for flow cytometry and CFU/MTT analyses due to sample availability

^†^ There was an interval of several years between the MRI scan and blood sampling for this study

Table abbreviations: *ATE* Arterial thromboembolism, *MRI* Magnetic resonance imaging, *NA* Not available, *TM* β thalassemia major, *VTE* Venous thromboembolism

### Hematopoietic-derived endothelial progenitor cells (hEPCs)

#### Quantity assessment of hEPCs

**Fig. 1:**
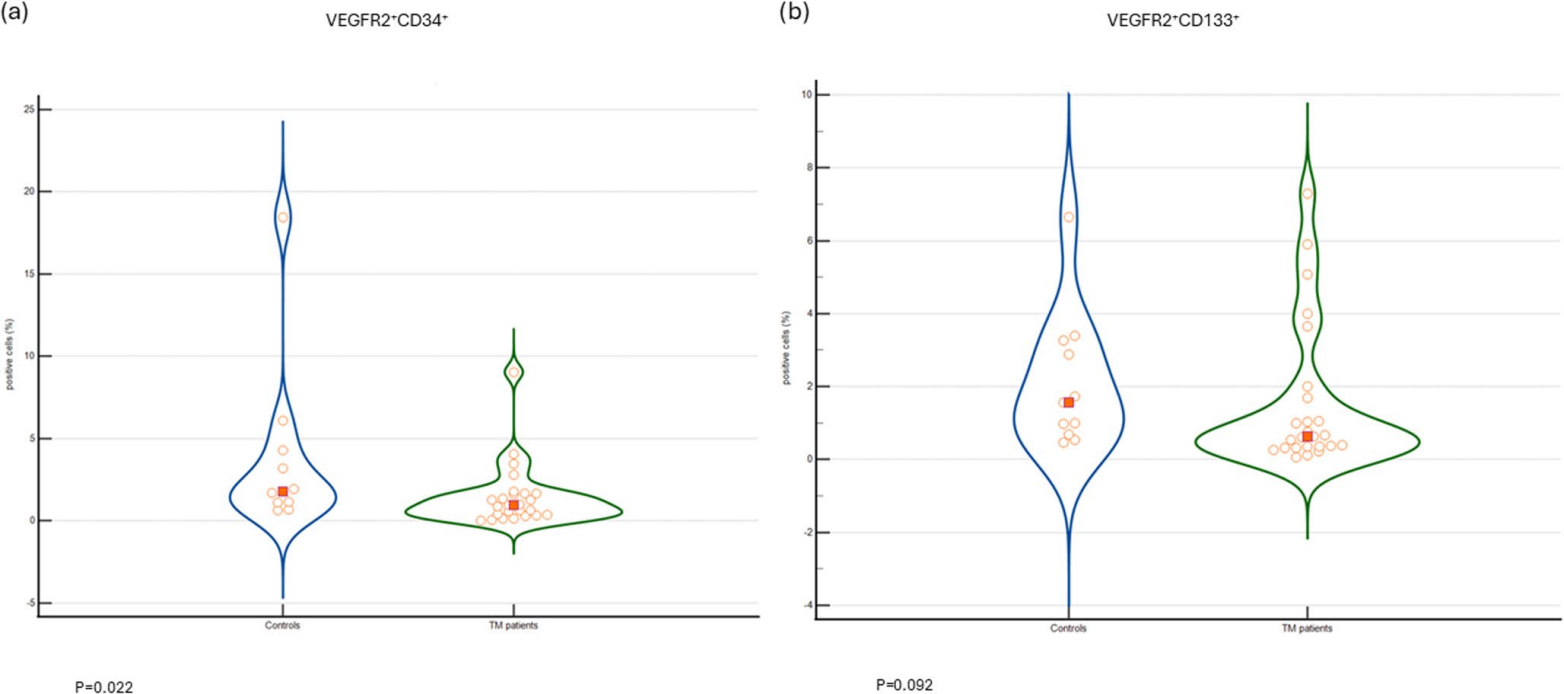
Violin plot representations of the proportion of (**a**) VEGFR-2^+^ CD34^+^ cells and (**b**) VEGFR-2^+^ CD133^+^ cells in TM patients (“TM”) (*n* = 25) vs. healthy controls (“Controls”) (*n* = 11)

TM patients had a significantly lower proportion of VEGFR-2^+^ CD34^+^ cells as compared to controls: 0.95% (0.36–1.65%) vs. 1.78% (1.1–4.28%), *p* = 0.02 (Fig. 1a). In addition, TM patients had a trend towards lower proportion of VEGFR-2^+^ CD133^+^ cells as compared to controls: 0.63% (0.36–1.7%) vs. 1.56% (0.68–3.27%), *p* = 0.09 (Fig. 1b).

#### Quality assessment of hEPCs

**Fig. 2:**
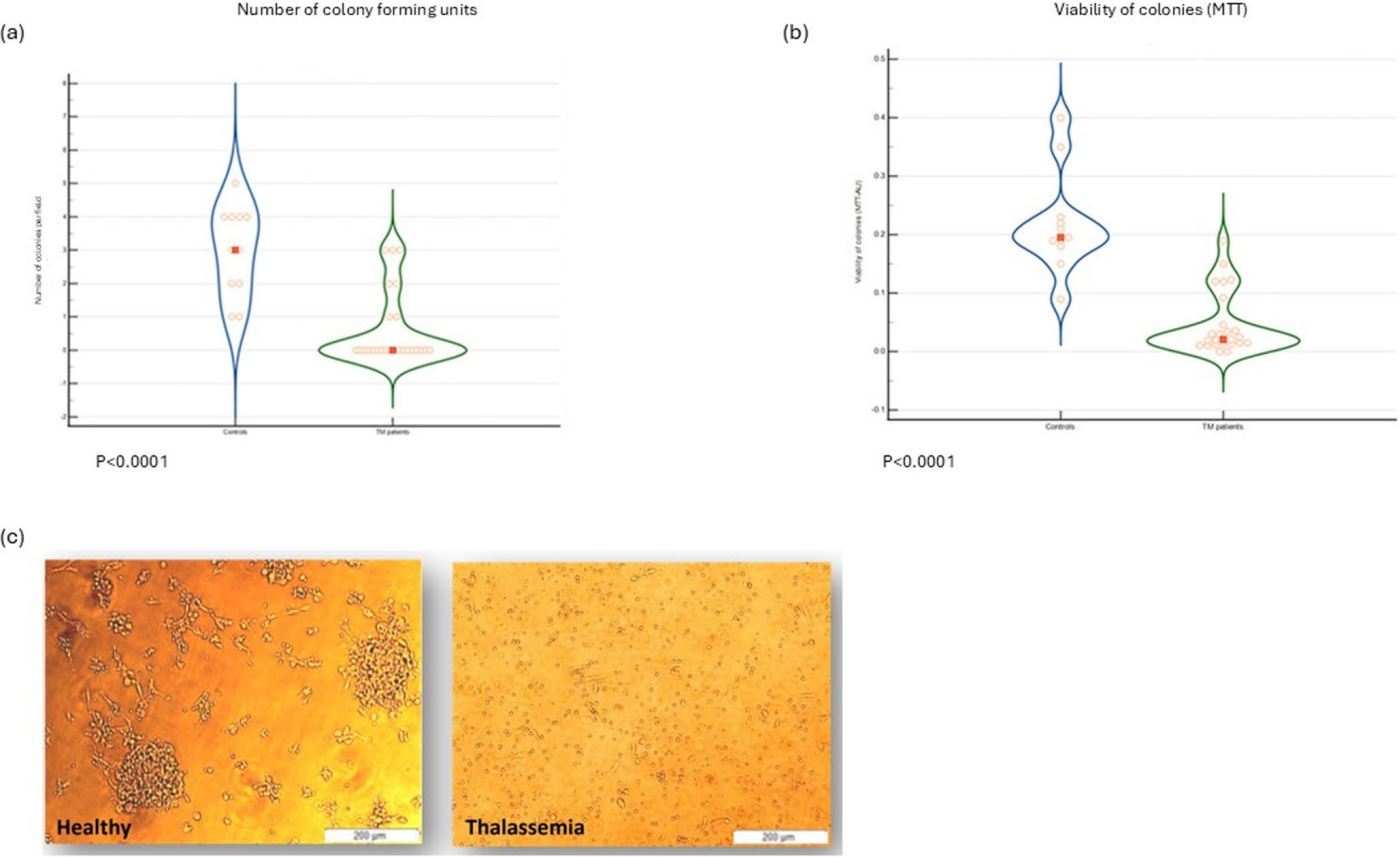
Violin plot representations of (**a**) the number of endothelial progenitor cells (EPC) colony-forming units (CFU) per field after 7 days of incubation in TM patients (*n* = 25) and in healthy controls (*n* = 11), (**b**) EPC viability assessed by MTT assay in TM patients (*n* = 25) vs. controls (*n* = 11), and (**c**) a representative image of CFUs derived from a TM patient and a healthy control. Abbreviations: AU = absorbance units

Following 7 days of incubation, the number of CFUs produced by TM patients was significantly lower compared to controls: 0.0 (0.0-0.5) vs. 3.0 (2.0–4.0), *p* < 0.0001 (Fig. 2a). Moreover, the viability of hEPCs was significantly reduced in TM patients compared to controls as demonstrated by MTT assay (OD 570 nm): 0.02AU (0.01–0.05) vs. 0.2AU (0.18–0.23), *p* < 0.0001 (Fig. 2b). A representative picture of hEPC colonies of a thalassemic patient and a control is presented in Fig. 2c.

#### Signaling molecules

There was no difference in plasma levels of VEGF and p-selectin between TM patients and controls (*n* = 15). However, there was a trend toward higher levels of SDF-1 among TM patients compared to controls: 3305pg/ml (2750-4475pg/ml) vs. 2805pg/ml (2380–3060pg/ml), respectively, *p* = 0.078.

### Mixing studies

Patients’ plasma did not inhibit the formation of hEPC CFUs obtained from healthy controls. The addition of patient plasma had no significant effect on either CFUs count or cell viability. Mean CFUs numbers were 3.66 ± 2.5 in regular media and 3.5 ± 2.29 in mixed thalassemia media. Mean MTT (OD 570 nm): 0.2AU (Absorbance Units) ± 0.017 in regular media compared to 0.19AU ± 0.017 in mixed thalassemia media.

### Subgroup analysis

#### Splenectomized (SPX) (*n* = 21) vs. non-splenectomized (non-SPX) (*n* = 4) patients

SPX patients had significantly lower proportions of VEGFR-2^+^ CD34^+^ cells, 0.64% (0.35–1.36%) versus 1.73% (1.47%-2.92%), as well as lower proportions of VEGFR-2^+^ CD133^+^ cells, 0.53% (0.32–0.99%) versus 3.54% (1.86%-5.49%) as compared to non-SPX patients. (*p* = 0.041 and *p* = 0.013, respectively).

Additionally, we performed a sensitivity analysis comparing non-SPX TM patients (*n* = 4) with controls. After 7 days of incubation, non-SPX patients demonstrated a significantly lower number of CFU 0.0 (0-0.5) vs. 3 (2–4) (*p* = 0.002) as well as significantly reduced viability of hEPCs 0.02AU (0.01–0.06) vs. 0.2AU (0.18–0.23) (*p* = 0.001) as compared to controls.

#### Previous thrombotic event (*n* = 6) vs. no history of thrombotic event (*n* = 19)

**Fig. 3:**
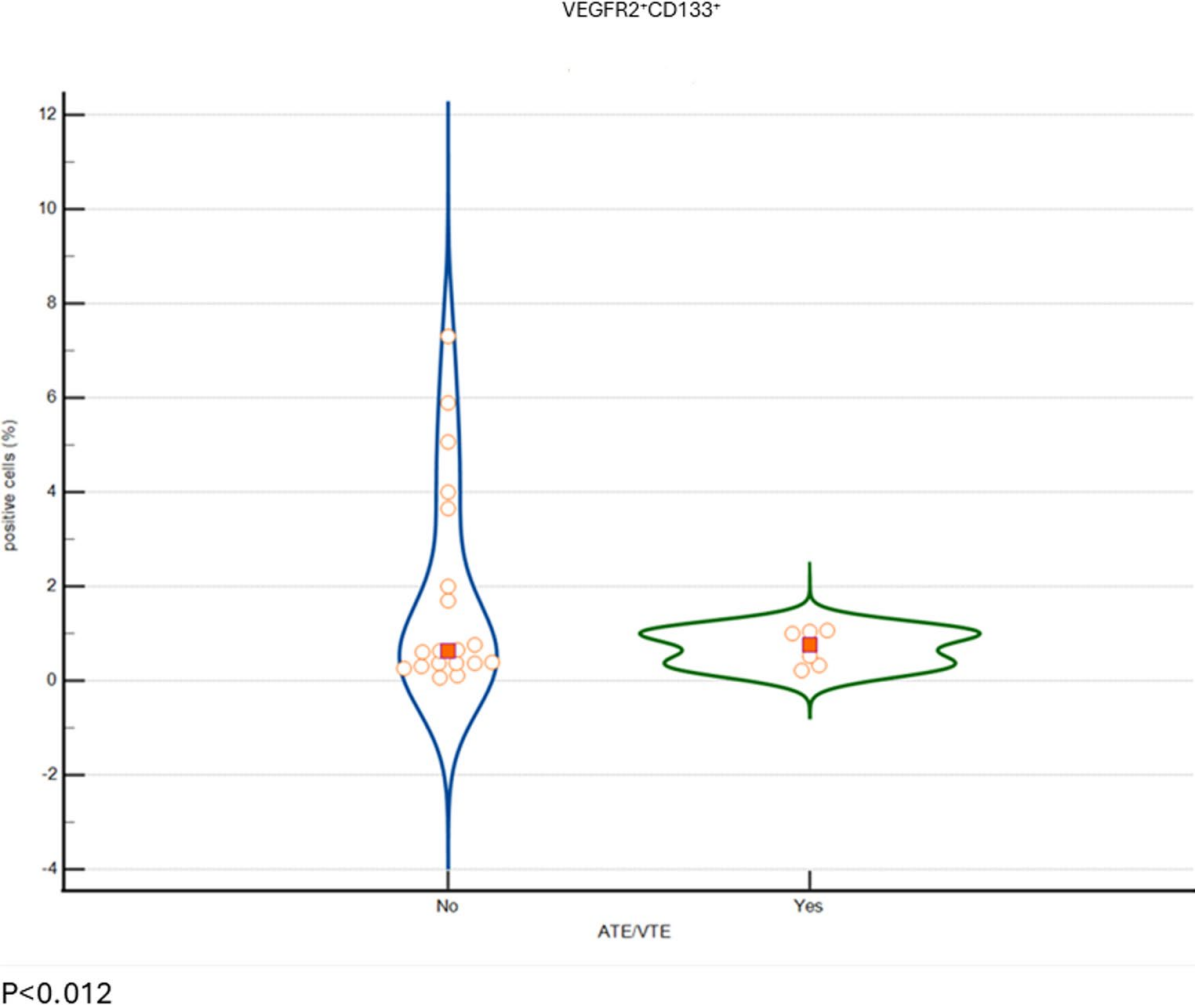
Violin plot showing the proportion of VEGFR-2^+^ CD34^+^ cells in TM patients with a history of thromboembolic events (*n* = 6) vs. TM patients with no history of thromboembolic events (*n* = 19)

Patients with a history of thrombotic events had a significantly lower proportion of VEGFR-2^+^ CD34^+^ cells, 0.36% (0.29–0.41%) compared to those with no history of thrombotic events, 1.29% (0.63–1.8%), *p* = 0.012. These results are shown in Fig. 3. We did not find a difference in the proportion of VEGFR-2^+^ CD133^+^ between these two subgroups (*p* = 0.68).

#### Ferritin level and type of chelation therapy

We did not find a significant difference in either the quantity or quality of hEPCs, nor in the level of signaling molecules (VEGF, p-selectin and SDF-1) between patients with high level of ferritin ( > = 1000micg/l, *n* = 13) and patients that reached the target level of ferritin (< 1000micg/l, *n* = 12) [38].

Subgroup analysis showed a significantly lower proportion of VEGFR-2^+^ CD34^+^ cells in patients treated with either DFO, 0.39% (0.18–0.52%), or DFP, 0.25% (0.1–0.49%), in comparison to patients treated with DFX, 1.28% (0.74–1.64%), (*p* = 0.016 and *p* = 0.011, respectively).

#### Silent cerebral ischemic (SCI) lesions in MRI

No correlation was observed between hEPC number, colony-forming capacity, or levels of signaling molecules (VEGF, p-selectin and SDF-1) and history of cerebral ischemic lesions as demonstrated by prior MRI studies (*p* = 0.74, *p* = 0.42, *p* = 0.44 for the difference in the proportion of VEGFR-2^+^ CD133^+^, VEGFR-2^+^ CD34^+^, and CFUs between patients with and without SCI lesions, respectively).

#### Antiplatelet and anticoagulant agents

To overcome the potential effect of antiplatelet and anticoagulant agents on hEPCs, we performed a sensitivity analysis including 16 TM patients that were not treated with anti-platelet or anticoagulation drugs (9 patients received one of these drugs at time of sampling were excluded). The proportions of both VEGFR-2^+^ CD34^+^ and VEGFR-2^+^ CD133^+^ cells were significantly lower among these patients, in comparison with controls (0.98% [0.47–1.51%] versus 1.78% [1.1–4.28%], *p* = 0.028, and 0.57% [0.32–1.39%] versus 1.56% [0.68–3.27%], *p* = 0.046, respectively). In addition, the amount of hEPCs measured by CFUs, 0.0 (0.0–0.0) and the viability of hEPCs measured by MTT, 0.02 (0.01–0.03), were significantly lower among these patients, in comparison with controls, 3.0 (2.0–4.0) and 0.2AU (0.18–0.23), (*p* < 0.001 and *p* < 0.001, respectively).

Subgroup Analyses of 25 patients with TM are shown in Table 2.

#### Sesitivity analysis excluding patients with diabetes

To account for the potential confounding effect of diabetes, we performed a sensitivity analysis including only non-diabetic TM patients (*n* = 21) and controls. Non-diabetic TM patients had a significantly lower proportion of VEGFR-2⁺ CD34⁺ cells compared with controls (0.95% [0.36–1.65] vs. 1.78% [1.1–4.28], *p* = 0.03). In addition, non-diabetic patients generated significantly fewer CFU colonies (0 [0–0] vs. 3 [2–4], *p* < 0.0001) and demonstrated reduced hEPC viability as assessed by the MTT assay (0.02 AU [0.01–0.04] vs. 0.2 AU [0.18–0.23]).

**Table 2:** Subgroup Analysis of 25 patients with TM

Parameter	Results*		*P* value
VEGFR-2^+^CD34^+^ (%)	SPX (*n* = 21) 0.64 (0.35–1.36)	No SPX (*n* = 4) 1.73 (1.47–2.92)	0.041
	Thrombosis (*n* = 6) 0.36 (0.29–0.41)	No thrombosis (*n* = 19) 1.29 (0.63–1.8)	0.012
	ICT–DFO (*n* = 4) 0.39 (0.18–0.52)	ICT–DFX (*n* = 8) 1.28 (0.74–1.64)	0.016
	ICT–DFP (*n* = 4) 0.25 (0.1–0.49)	ICT–DFX (*n* = 8) 1.28 (0.74–1.64)	0.011
VEGFR-2^+^CD133^+^ (%)	SPX 0.53 (0.32–0.99)	No SPX 3.54 (1.86–5.49)	0.013
	Thrombosis 0.76 (0.32–1.04)	No thrombosis 0.63 (0.36–3.65)	0.679
	ICT–DFO 0.66 (0.27–1.02)	ICT–DFX 0.71 (0.38–2.83)	0.445
	ICT–DFP 0.32 (0.19–0.45)	ICT–DFX 0.71 (0.38–2.83)	0.074
EPC’s CFUs count	SPX 0 (0-0.5)	No SPX 0 (0-0.5)	0.779
	Thrombosis 0 (0–0)	No thrombosis 0 (0–1)	0.629
	ICT–DFO 0 (0–0)	ICT–DFX 0 (0-0.75)	0.220
	ICT–DFP 0 (0–1)	ICT–DFX 0 (0-0.75)	0.84
EPC viability MTT (AU)	SPX 0.03 (0.02–0.05)	NO SPX 0.02 (0.01–0.06)	0.504
	Thrombosis 0.02 (0.01–0.03)	No thrombosis 0.02 (0.01–0.09)	0.679
	ICT–DFO 0.01 (0.01–0.02)	ICT–DFX 0.03 (0.02–0.07)	0.173
	ICT–DFP 0.02 (0.02–0.08)	ICT–DFX 0.03 (0.02–0.07)	0.799
VEGF (pg/ml)	SPX 135.5 (55-1019.5)	No SPX 377.5 (46-758.5)	0.578
	Thrombosis 199.2 (122–758)	No thrombosis 116 (46-1019.5)	0.545
	ICT–DFO 199.2 (102.2-879.5)	ICT–DFX 435.2 (33.2-1003.5)	0.671
	ICT–DFP 437 (92-1173.2)	ICT–DFX 435.2 (33.2-1003.5)	0.552
P-selectin (ng/ml)	SPX 157.4 (130.7-219.5)	No SPX 107.4 (77.3-136.6)	0.157
	Thrombosis 137.8 (123.5-225.9)	No thrombosis 158.8 (130.4-211.4)	0.861
	ICT–DFO 174.7 (121.3-239.5)	ICT–DFX 154.6 (118.9-230.4)	0.94
	ICT–DFP 143.2 (130.9-211.4)	ICT–DFX 154.6 (118.9-230.4)	0.779
SDF-1 (pg/ml)	SPX 2885 (2620–4015)	No SPX 4642.5 (4190–4895)	0.065
	Thrombosis 2772.5 (2260–4015 )	No thrombosis 3380 (2750–4530)	0.616
	ICT–DFO 3402.5 (2772.5-5002.5)	ICT–DFX 4397.5 (3280–4810)	0.862
	ICT–DFP 2970 (2505-3247.5)	ICT–DFX 4397.5 (3280–4810)	0.63

^*^ All values are in median (IQR)

Table abbreviations: *CFU* Colony forming units, *DFO* deferoxamine, *DFP* deferiprone, *DFX* deferasirox, *EPC* Endothelial progenitor cells, *ICT* Iron chelation therapy, *SDF-1* stromal cell-derived factor 1, *SPX* Splenectomized, *VEGF* Vascular endothelial growth factor

## Discussion

In this study, we have shown that TM patients had a significantly lower proportion of hEPCs in peripheral blood in comparison with healthy controls. In addition, hEPCs derived from TM patients produced lower number of CFUs. To our knowledge, only two prior studies have evaluated VEGFR-2^+^ CD34^+^ cells in patients with TM [35, 39]. In contrast to our findings, both reported higher proportions of these cells in TM patients compared with controls. While the basis for this discrepancy remains uncertain, we noticed an age difference which could potentially explain these results. Adly et al. focused on pediatric TM patients, and Cheung et al. examined a relatively young cohort (mean age 26.9 years), approximately a decade younger than our cohort [35, 39]. Notably, despite reporting higher circulating hEPC counts, Cheung et al. also observed impaired colony-forming capacity in TM patients, a functional deficit that closely aligns with our findings.

The reduced colony number in TM patients could theoretically reflect a lower circulating hEPC number. However, the complete absence of colonies (median number of colonies was zero) is unlikely to be fully explained by reduced number of progenitor cells alone and may suggest impaired colony-forming capacity rather than a purely quantitative defect. To minimize the possibility that differences in seeding viability contributed to these findings, cell viability was confirmed prior to plating using Trypan Blue exclusion. Consistent with this interpretation, while the MTT assay reflects metabolic activity and may be influenced by cell number, it was used as a supportive measure rather than a standalone functional assessment, and should be interpreted within this context.

Patients with thalassemia are known to have increased reactive oxygen species (ROS) [20, 39]. The pronounced oxidative stress characteristics of TM are driven by several interconnected processes, such as iron accumulation, ineffective erythropoiesis, and the presence of excess unpaired α-globins [40–42]. It was shown that excessive production of ROS inhibits nitric oxide (NO) production and results in inhibition of the mobilization, proliferation, and differentiation of hEPCs [43, 44]. We therefore assume that the decreased number and impaired colony-forming capacity of hEPCs among patients with TM may be attributed to the high oxidative stress among these patients. The mixing study results do not support this mechanism, as plasma derived from TM patients did not inhibit hEPC CFUs formation in cells derived from healthy controls. Several theoretical explanations are possible: First, we did not assess the effect of mixing plasma on the quantity of hEPCs. Second, it is possible that a larger concentration or longer exposure time are needed to demonstrate the oxidative effect on hEPCs. Third, other intrinsic cellular mechanisms of the hEPCs might be involved.

We showed a significantly lower proportion of VEGFR-2^+^ CD34^+^ and VEGFR-2^+^ CD133^+^ cells among SPX TM patients. These results echo the conclusions of previous studies which have suggested that the spleen is a major reservoir of hEPCs during their circulation and plays a role in the modulation of circulating hEPC kinetics [45]. In a sensitivity analysis, after 7 days of incubation, hEPCs derived from non-SPX TM patients produced a significantly lower number of CFUs and exhibited significantly lower viability compared to hEPCs derived from healthy controls. Although the non-SPX subgroup is small, these results support the notion that the reduced number and impaired colony-forming capacity of hEPC derived from TM patients may primarily reflect disease-related mechanisms and are not necessarily dependent on splenectomy status.

Among viable measures, serum ferritin is the standard marker for evaluating systemic iron burden [3, 46]. Biologically active iron species, including labile plasma iron and non-transferrin-bound iron (NTBI) have been implicated in endothelial injury [47, 48]. In our previous study [11] we showed a significant association between iron overload, as reflected by high level of serum ferritin, and the presence of SCI. In the present study, however, no significant correlations were observed between serum ferritin levels and either the quantity or quality of hEPCs, or the concentrations of signaling molecules (VEGF, p-selectin and SDF-1). Consistent with our findings, Cheung et al. also reported no correlation between serum ferritin levels and the number of CFUs [35]. The lack of association may be biologically plausible, since ferritin has recognized limitations as a surrogate marker of biologically active iron, whereas vascular injury is thought to be driven primarily by labile plasma iron and NTBI rather than total iron stores [49]. Furthermore, hEPC impairment in TM may be mediated by additional mechanisms such as chronic inflammation, oxidative stress, and hemolysis-related endothelial dysfunction. These mechanisms are not necessarily reflected by ferritin levels.

In a subgroup analysis we found that TM patients treated with either DFP or DFO had a significantly lower proportion of VEGFR-2^+^ CD34^+^ cells compared to patients that were treated with DFX. Yet, to the best of our knowledge, neither of these chelating agents is known to be associated with increased thrombotic risk. Given the small size of these subgroups within our cohort, further validation in larger patient populations is warranted.

Depletion or dysfunction of hEPCs contributes to endothelial impairment and plays a key role in the pathogenesis of cardiovascular diseases [50]. In addition, there is evidence for the protective role of hEPCs in venous thromboembolism [29, 32, 33]. CD34 and CD133 (stem cells markers) co labeled with VEGFR-2 (a receptor tyrosine kinase on endothelial cells) are widely used markers to define putative circulating EPCs, whose expression is thought to reflect bone marrow mobilization and involvement in vascular repair processes. Complementing these progenitor cell markers, SDF-1 is a key chemokine regulating hEPCs trafficking and homing to sites of endothelial injury, thereby facilitating endothelial regeneration [51]. In our cohort, hEPC levels were significantly reduced, while circulating SDF-1 levels showed a non-significant trend toward higher values. Although this difference did not reach statistical significance, it may reflect a compensatory upregulation of SDF-1 in response to impaired hEPC availability or function, aimed at enhancing progenitor cell recruitment.

Reduced number or impaired colony-forming capacity of hEPCs may indicate ineffective endothelial repair and are linked to an increased risk of arterial and venous thrombosis. It was also shown that vascular damage progression correlated with hEPC count [52]. In a subgroup analysis, we have shown a significantly lower proportion of VEGFR-2^+^ CD34^+^ cells among TM patients with a history of either arterial or venous thrombotic events, in comparison to TM patients without a history of thrombotic event. This finding supports the protective role of hEPCs against thrombotic events in TM patients.

Moreover, CD34^+^ Cells and hEPC subpopulations are known to be associated with cerebral small vessel disease burden [53]. However, we did not find a correlation between a decrease in the number or colony-forming capacity of hEPCs and the occurrence of ischemic brain lesions, as demonstrated by MRI studies. The mechanism for these lesions needs further research and is presumed to be multifactorial involving hypercoagulable state, damaged red blood cells and toxicity of free iron species [11, 16, 54].

Anti-platelet and anticoagulant agents may influence the number and function of hEPCs. In-vitro exposure to acetylsalicylic acid was associated with reduced number and impaired function of hEPCs [55]. In contrast, rivaroxaban was associated with increased differentiation ability of hEPCs [56]. To overcome this effect, we performed a sensitivity analysis by excluding all patients treated with either antiplatelet or anticoagulant agent. In this analysis, TM patients differed significantly from healthy controls in both the number and colony-forming capacity of hEPCs. These findings indicate that such reductions in hEPCs count and colony-forming capacity persist in TM patients, independent of any influence from antiplatelet and anticoagulant therapy.

### Study limitations and strengths

Regarding the constraints of this study, the limited sample size stands out as the most significant, with the greatest implications for the subgroup analyses. Therefore, the findings from the subgroup analyses should be regarded as exploratory and require confirmation in larger cohorts. Diabetes was presented in a minority of patients (16%) and may represent a potential confounder given its known effect on hEPC biology. However, the observed prevalence is consistent with previously reported rates in thalassemia cohort [57]. Importantly, in a sensitivity analysis excluding patients with diabetes, the differences in CD34⁺VEGFR-2⁺ cell counts, CFU number, and CFU viability between TM patients and controls remained statistically significant, supporting the robustness of our findings. Additionally, While the culture conditions enrich for endothelial progenitor cells, the CFU assay may include both hematopoietic and endothelial progenitors, which should be considered when interpreting the results. In addition, CFU assays were performed using a fixed PBMC input without normalization to EPC counts; therefore, a contribution of reduced progenitor cell input to the lower colony numbers cannot be entirely excluded, although the frequent absence of colonies suggests an impairment beyond purely quantitative differences. Our study has several strengths; We examined both quantity and quality of hEPCs. The results obtained from different tests were consistent and demonstrated both reduced quantity and impaired colony-forming capacity of hEPCs among TM patients. Yet, despite the relatively small study group, we presume that our results reliably indicate a true phenomenon as they were consistent. Larger studies are needed to confirm our results and to assess the different factors that affect hEPCs specifically in TM patients. 

### Conclusions

In conclusion, the present study demonstrates that a reduced number and impaired colony-forming capacity of hEPCs are common in adult TM patients and may represent one of several factors contributing to their hypercoagulable state.

## Data Availability

The dataset generated and analyzed during the current study are not publicly available due to the small sample size and institutional privacy restriction. However, datasets are available from the corresponding author on a reasonable request.
